# The Effecting Mechanisms of 100 nm Sized Polystyrene Nanoplastics on the Typical Coastal *Alexandrium tamarense*

**DOI:** 10.3390/ijms25137297

**Published:** 2024-07-02

**Authors:** Luying Li, Qian Liu, Bo Li, Yan Zhao

**Affiliations:** 1 Marine Science and Technology College, Zhejiang Ocean University, Zhoushan 316022, China; liluying7867@stu.ouc.edu.cn; 2 Department of Marine Ecology, College of Marine Life Sciences, Ocean University of China, Qingdao 266003, China; zhaoyan@ouc.edu.cn; 3 Marine Science Research Institute of Shandong Province, Qingdao 266104, China; raulliuqian@163.com; 4 Qingdao Key Laboratory of Coastal Ecological Restoration and Security, Qingdao 266104, China

**Keywords:** nanoplastics, *Alexandrium tamarense*, physiological–biochemical performance, gene information, effecting mechanisms

## Abstract

Due to the increase in nanoplastics (NPs) abundance in aquatic environments, their effects on phytoplankton have aroused large research attention. In this study, 100 nm sized polystyrene NPs were chosen to investigate their effecting performance and mechanisms on a typical dinoflagellates *Alexandrium tamarense*. The results indicated the population growth and photosynthetic efficiencies of *A. tamarense* were significantly inhibited by NPs exposure, as well as the increase in cellular total carotenoids and paralytic shellfish toxins (PSTs). Meanwhile, the cellar ROS levels increased, corresponding to the increased activities or contents of multiple antioxidant components, including SOD, CAT, GPX, GR, GSH and GSSG. The transcriptional results support the physiological–biochemical results and further revealed the down-regulation of genes encoding the light reaction centers (PSI and PSII) and up-regulation of genes encoding the antioxidant components. Up-regulation of genes encoding key enzymes of the Calvin cycle and glycolytic pathway together with the TCA cycle could accelerate organic carbon and ATP production for *A. tamarense* cells resistant to NPs stress. Finally, more Glu and acetyl-CoA produced by the enhanced GSH cycle and the glycolytic pathway, respectively, accompanied by the up-regulation of Glu and Arg biosynthesis genes supported the increase in the PST contents under NPs exposure. This study established a data set involving physiological–biochemical changes and gene information about marine dinoflagellates responding to NPs, providing a data basis for further evaluating the ecological risk of NPs in marine environments.

## 1. Introduction

Nowadays, the presence of plastic particles in the marine ecosystem has become one of the most worrying pollution problems. Plastic particles with a size of 1–5 mm are defined as microplastics (MPs), while plastic particles < 1 μm are defined as nanoplastics (NPs) [1]. A continual increase in micro-nanoplastics (MNPs) abundance was observed in marine environments, and the maximum concentration of MNPs could reach up to 6.981 μg L^−1^ in the world’s oceans [2]. Isobe et al. (2019) [3] predicted MNPs concentrations could reach up to an mg L^−1^ level in 2066 based on numerical modeling simulations. As smaller particles than MPs, NPs are more likely to be ingested by marine organisms through predation, even respiration, but are more difficult to be expelled from organisms [4]. Moreover, NPs have a higher specific surface area than MPs, leading to a higher adsorption potential for other pollutants including heavy metals and persistent organic pollutants (POPs) [5]. Very few studies specifically investigated the environmental concentrations of NPs. Ter Halle et al. (2017) [6] measured the distribution of NPs in the North Atlantic Subtropical Gyre and showed that the concentration of NPs ranged from 13 to 501 NPs m^−3^.

The nanotoxicological research about NPs on marine organisms has been started, and most studies about metazoan indicated NPs were more toxic than MPs due to higher ingestion rates, more intestinal damage and higher aggregation potential [7,8]. Phytoplankton are the base of the aquatic food web, which play important roles on the material cycle and energy flow in marine ecosystems. Previous studies have proved that MNPs could cause toxicities on phytoplankton and pointed out the toxicities could be increased due to the decrease in the particle size. For example, Sjollema et al. (2016) [9] observed a more severe inhibition of *Dunaliella tertiolecta* growth caused by uncharged 0.5 μm than 6 μm PS beads. Chae et al. (2019) [10] indicated that the effect of MNPs on microalgae could change from positive to negative when the cell/MNPs size ratio was approximately decreased by 0.75–3.07. Gao et al. (2020) [11] summarized that different-sized PS MNPs from 0.055 to 72 μm could induce the half effecting concentration (EC_50_) values for microalgae from high to low. However, when comparing 100 nm NPs and 1 μm MPs, some studies found 1 μm MPs was more toxic than 100 nm NPs for *Thalassiosira pseudonana*, *Scenedesmus obliquus* and *Microcystis aeruginosa* [12,13,14]. Moreover, the effecting mechanisms of NPs were also different from MPs, which were more easily adsorbed by microalgal cells and caused photosynthetic inhibition, as well as more easily aggregating with microalgal cells and causing enhanced settlement rates [13,14].

Much fewer studies about NPs affecting microalgae have been conducted than MPs; thus, more comprehensive research is required. The commonly detected MNPs in the marine environment are mainly polypropylene (PP), low-density polyethylene (LDPE), high-density polyethylene (HDPE), polymethyl methacrylate (PMMA), polystyrene (PS), polyvinyl chloride (PVC), polyurethane (PUR) and polyethylene terephthalate (PET), in which those low-density MNPs such as PE and PS MNPs (density < 1.02–1.07 g cm^−3^) could float and accumulate on the sea’s surface [1]. Due to the enhancement in seawater eutrophication and shift in nutrient structure, dinoflagellates have become the most abundant phytoplankton group in many coastal regions, sometimes exceeding the abundance of diatoms [15]. Dinoflagellates are known to cause harmful algal blooms (HABs), and *Alexandrium* is one of the most widespread distributed dinoflagellates genera that frequently cause harmful algal blooms (HABs) in coastal areas [16]. A high appearance of MNPs was also detected in coastal areas that interact with dinoflagellates, especially floating ones; thus, the effects of NPs on dinoflagellates require research attention. Therefore, we chose the common floating PS NPs and common dinoflagellates *Alexandrium tamarense* as the research target, comprehensively investigated the toxic effects of 100 nm NPs on *A. tamarense* with respect to population growth, photosynthesis, pigments, oxidative stress and toxins production, and further elucidated the underlying molecular toxic mechanisms on the transcriptional level. This study could establish a complete data set for NPs affecting marine dinoflagellates, providing an important basis for evaluating the ecological hazards of NPs in coastal environments.

## 2. Results and Discussion

### 2.1. Effects of NPs on the Growth and Photosynthetic Changes of A. tamarense

Five mg L^−1^ 100 nm PS NPs significantly inhibited the population growth and photosynthetic efficiencies of *A. tamarense*, especially in the first 48 h (*p* < 0.05, Table 1). The relative growth rates of the NPs group were lower than the controls until 144 h, and the maximum quantum yields of PSII (Fv/Fm) were less sensitive than the maximum electron transportation rates (rETR_max_) responding to NPs (Table 1). Cellular chlorophyll (chl *a*) did not show much difference under NPs stress, while the total carotenoid contents increased by 21.3% and 27.6% at 96 h and 144 h, respectively (*p* < 0.01, Table 1). Many previous studies have shown that population growth and PS II photosynthetic parameters were proper biomarkers for microalgae responding to environmental stress, which also fitted for our results [17]. An increased total carotenoid contents in the late experimental stage indicated a positive stress response due to the antioxidant functions of some carotenoids, such as β-carotenoids [18].

### 2.2. Effects of NPs on the Antioxidant Component Changes of A. tamarense

**Figure 1:**
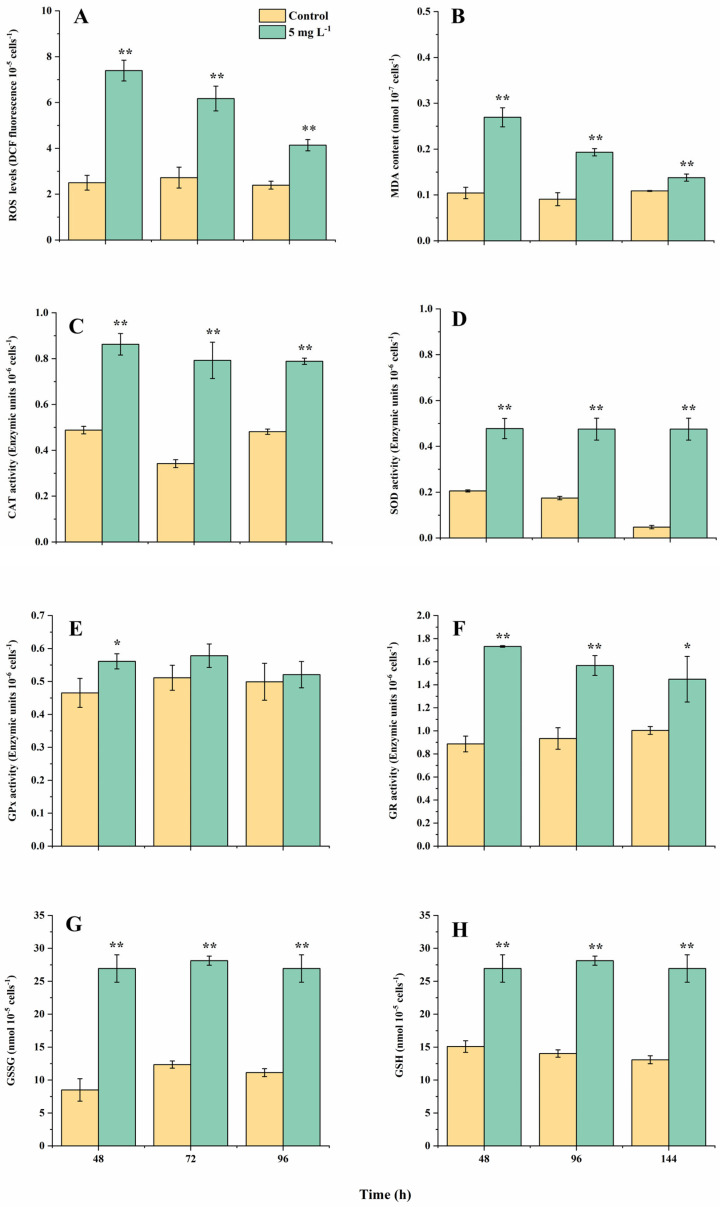
The changes in intracellular reactive oxygen species (ROS) levels (**A**), malondialdehyde (MDA) contents (**B**), activities of catalase (CAT) (**C**), superoxide dismutase (SOD) (**D**), glutathione peroxidase (GPx) (**E**) and glutathione reductase (GR) (**F**), oxidized glutathione (GSSG) (**G**) and glutathione (GSH) (**H**) contents in *A. tamarense* cells in different treatments at 48, 96 and 144 h. Data are shown as mean ± S.D. * indicates a statistical significance compared to the controls at *p* < 0.05 level, ** indicates *p* < 0.01 level.

The cellular reactive oxygen species (ROS) level, malondialdehyde (MDA, indicating the oxidative state of cell membrane) contents, catalase (CAT, converting H_2_O_2_ to H_2_O) and superoxide dismutase (SOD, converting O_2_^−^ to H_2_O) activities all showed a significant increase under NPs exposure (Figure 1A–D). The ROS levels in the NPs group peaked at 48 h while they were still 73.2% higher than the controls at 144 h, consistent with the changing trend of the MDA contents (Figure 1A,B; *p* < 0.01). The decrease in the ROS and MDA contents with the culturing time indicated increased cell antioxidant abilities, corresponding to the recovery of population growth and photosynthetic efficiencies, which could be attributed to the activation of the antioxidant system. The two key antioxidant enzymes showed consistently high levels from 24 h to 144 h (*p* < 0.01). The SOD and CAT activities maximally increased by 879.2% and 132.4% in the NPs treatment at 96 and 144 h, respectively (Figure 1C,D; *p* < 0.01).

Correspondingly, changes in the components in the glutathione (GSH) cycle also happened. The glutathione (GSH) and oxidized glutathione (GSSG) contents in the NPs treatment both increased throughout the experiment, which were 78.6–106.8% and 97.1–216.9% higher than the controls, respectively (Figure 1G,H; *p* < 0.01). Glutathione reductase (GR) is responsible for converting GSSG to GSH, whose activities were 94.4% and 68.8% higher than the controls at 48 and 96 h (*p* < 0.05). On the contrary, the activities of glutathione peroxidase (GPx, converting GSH to GSSG) only showed an obvious increase under the NPs exposure at 48 h (increased 20.4%; *p* < 0.05), indicating the GSH cycle tended to enhance the synthesis of GSH to scavenge the excess cellular ROS. Among all the measured physiological–biochemical parameters, only the relative growth rate and rETR_max_ values showed a significant difference with the controls at 48 h among all the relevant growth and photosynthetic parameters, while all the relevant cellular oxidative parameters showed a significant increase at 48 h. Since the cellular ROS levels were easier to detect than the other oxidative stress parameters, we considered the relative growth rate, rETR_max_ and ROS as proper biomarkers for further toxicological assessments.

### 2.3. Effects of NPs on Cellular PST Production of A. tamarense

**Figure 2:**
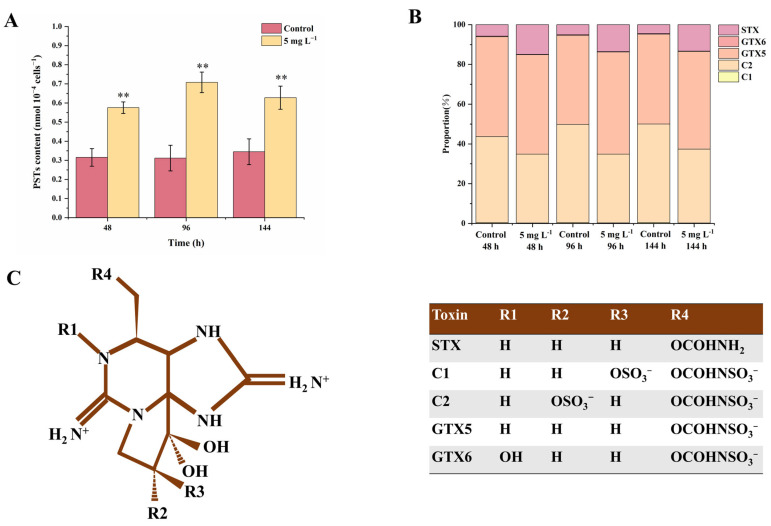
The contents (**A**), composition (**B**) and chemical structure (**C**) of cellular paralytic shellfish toxins (PSTs) in *A. tamarense* cells of different groups at 48, 96 and 144 h. Data are shown as mean ± S.D. ** indicates a statistical significance compared to the controls at *p* < 0.01 level. Chemical structure of detected PSTs in this experiment were modified from Wang et al. (2005) [24].

The celluar paralytic shellfish toxins (PST) contents were significantly increased by NPs exposure, which increased by 82.7%, 129.0% and 85.3% at 48, 96 and 144 h compared with the controls (*p* < 0.01). Five kinds of PSTs including C1, C2, GTX5, GTX6 and STX were detected in the *A. tamarense* cells (the full name and chemical structure of the five PSTs are shown in Figure 2C), in which C1 and C2 accounted for more than 34.8% of the total PST contents. The relative composition was also changed by NPs, showing an increase in the STX proportion and a decrease in the C2 proportion. The proportions of STX increased from 4.6–5.8% in the controls to 13.3–15.0% in the NPs group *(p* < 0.01), while the proportions of C2 decreased from 43.3–50.0% in the controls to 34.6–37.1% in the NPs group (*p* < 0.01). The proportions of GTX5 also increased under NPs exposure but without a statistically significant difference (*p* > 0.05). Li et al. (2023) [19] proved that the increase in PSTs under 1 μm PS MPs was caused by elevated ROS levels, which induced the enhanced biosynthesis of glutamate (Glu). Glu is the precursor for Arginine (Arg), and Arg participates in the first step of PST biosynthesis (Claisen condensation) [20]. Glu is another non-enzymatic antioxidant material and could be transformed to GSH, corresponding to the increased GSH contents in our results [21]. The next step following the Claisen condensation is the synthesis of STX, then STX could be transformed to C1 and C2 by a series of reductions or sulfonation reactions [22]. Therefore, the cellular oxidative state could block the reduction in STX to C1 and C2, leading to the increase in STX proportions and decrease in C2 proportions (Figure 2). Among all the PST components, STX was more toxic than other toxins; thus, NPs exposure not only increased the PST production, but also increased the overall toxicity of PSTs [23].

### 2.4. RNA Sequencing in A. tamarense under NPs Exposure

**Figure 3:**
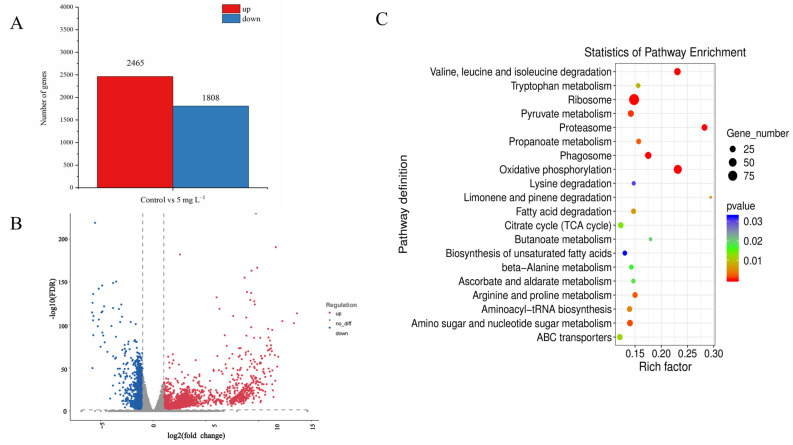
Transcriptomic analysis of *A. tamarense* in the control group and the 5 mg L^−1^ NPs group at 48 h. (**A**) The column chart of the number of statistical DEGs. (**B**) The volcano plot displays the distribution of DEGs. (**C**) Top 20 enrichment KEGG pathways.

After 48 h of NPs exposure, approximately 511,600–282,800 sequenced fragments (reads) were generated in the samples of the control and 5 mg L^−1^ NP-treated *A. tamarense* by RNA sequencing (Table S1). The de novo assembly results showed good quality of transcriptome assembly for our samples (Table S2). According to the threshold FDR < 0.05 and |log_2_ Fold Change| > 1, 2465 differential expressed genes (DEGs) were identified between the control and the NPs treatment, in which 2465 DEGs were up-regulated and 1808 DEGs were down-regulated (Figure 3A,B). To further analyze the functions of the DEGs, the DEGs were analyzed by the KEGG database, and the results showed that the DEGs were mainly enriched in pathways including proteasome, glycolysis/gluconeogenesis, TCA cycle, etc. (Figure 3C). Together with those significant physiological–biochemical changes, we also analyzed the gene expression changes regarding photosynthesis, the antioxidant system and PST synthesis, to further investigate the molecular effecting mechanisms of PS NPs on *A. tamarense* cells.

### 2.5. Decreased Photosynthetic Efficiency and Enhanced Pigment Synthesis in A. tamarense under NPs Exposure

**Figure 4:**
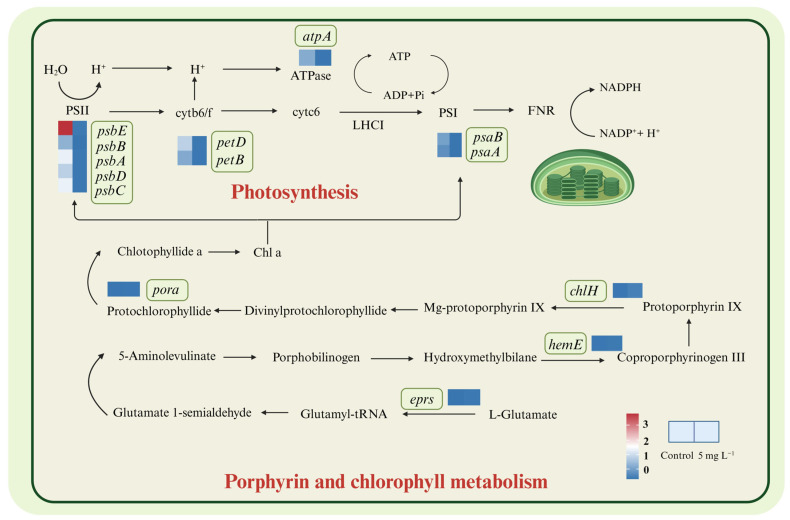
DEGs involved in the photosynthesis, porphyrin and chlorophyll metabolism in *A. tamarense* under PS NPs exposure.

Photosynthesis plays the most fundamental functions for microalgal cell divisions, and most key genes encoding PS II and PS I showed a down-regulated trend. Genes (*psbA*, *psbB*, *psbC*, *psbD* and *psbE*) encoding PS II key subunits were all significantly down-regulated (Figure 4, Table S3). Microalgal cells could repair damaged PSII proteins under stress conditions by replacing damaged proteins with newly synthesized subunits, while our results indicated *A. tamarense* cells did not activate this mechanism under NPs exposure [25]. Genes encoding the photosynthetic reaction center proteins A1 (*psaA*) and A2 (*psaB*), which comprise the PS I complex, also showed significant down-regulation under NPs exposure (Figure 4). The inhibition of PS I and PS II functions could lead to the slowdown of the linear and cyclic electron flow and reduced the transition from NADP to NADPH [26]. The cytochrome b6/f complex participates in the H^+^ transmembrane transport process through the cell membrane, which is able to transfer electrons from PSII to PSI [27], whose encoding genes *petB*, *petD* also showed down-regulation (2^−4.66^, 2^−4.84^) under NPs exposure. Additionally, the gene (*atpA*) encoding the α subunit of F-type ATPase was down-regulated, which also indicated the inhibition of the H^+^ transmembrane transport under NPs exposure. Overall, the down-regulation of genes encoding PSI, PSII, cytochrome b6/f complex and F-type ATPase indicated the inhibition of light reactions in chloroplasts, corresponding to the decrease in PSII photosynthetic efficiencies.

Regarding the pigments synthesis, some genes encoding enzymes of the chl *a* synthesis pathway were significantly up-regulated, such as *chlH* encoding the Mg chelatase, *pora* encoding NADPH protochlorophyllate oxidoreductase, *hemeE* encoding uroporphyrinogen decarboxylase and *eprs* encoding glutamyl-tRNA synthetase, in which *chlH* up-regulated the most (2^2.41^) under NPs exposure compared to the controls (Figure 4 and Table S3) The up-regulation of *chlH* could cause the enhancement in Mg chelatase synthesis, leading to the accumulation of photoporphyrin IX. Tarahi Tabrizi et al. (2016) [28] found the accumulation of photoporphyrin IX could be excited by light and transfer its excitation energy to O^2^, forming single-linear oxygen (^1^O_2_) then accelerating the chloroplast ROS production, corresponding to the results of elevated ROS levels under NPs exposure (Figure 1). Su et al. (2022) [29] also indicated that biodegradable MPs stimulated the cellular pigments increase in *Chlorella vulgaris*, as well as the up-regulation of genes encoding the chlorophyll synthesis pathway.

### 2.6. Antioxidant Defense System and Proteasome Pathway of A. tamarense Were Activated under NPs Exposure

**Figure 5:**
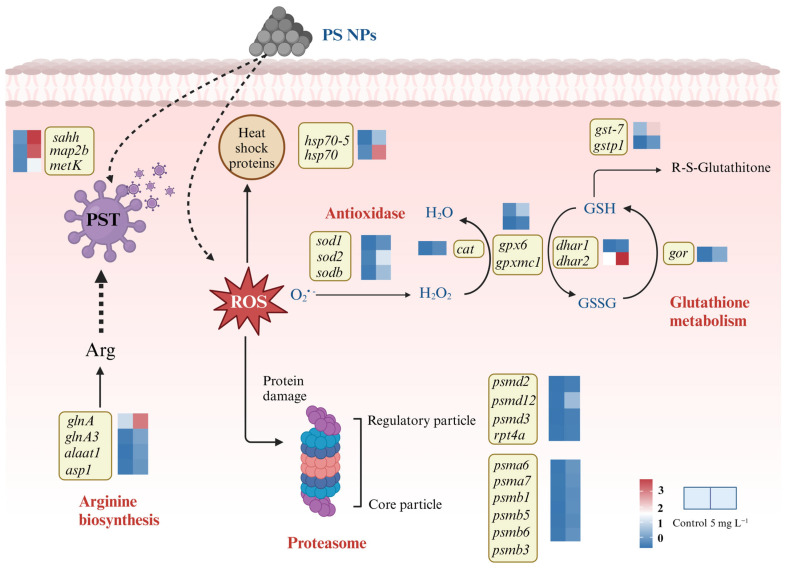
DEGs involved in the antioxidant system and proteasome pathway in *A. tamarense* under PS NPs exposure.

Enhanced antioxidant activities were observed in *A. tamarense* under NPs exposure, and more information could be explored based on the transcriptomic analysis (Figure 5). Firstly, the SOD-encoding genes *sod1*, *sodb* and *sod2* were up-regulated 2^3.21^-, 2^2.75^- and 2^2.91^-folds under NPs exposure, respectively, and the CAT-encoding gene (*cat*) was up-regulated 2^3.75^-fold, indicating the enhancement in SOD and CAT biosynthesis, corresponding to the increased SOD and CAT activities on the biochemical level (Figure 1). Secondly, genes involving the GSH cycle were up-regulated, such as *gpx6* and *gpxmc1* (encoding GPX), *dhar1* and *dhar2* (encoding dehydroascorbate reductase; DHAR), *gor* (encoding GR), *gst7* and *gstp1*(encoding GST). Combined with the biochemical changes, the logic of the GSH cycle changes could be concluded as follows: enhanced GR activity could produce more GSH, which could scavenge ROS due to the increase in GPX and GST activities. The turnover rates between GSH and GSSG could be enhanced by increased GR, DHAR and GPX activities.

It is worth mentioning that the product of GSH catalyzed by GST is Glu, which is the precursor of the key raw material Arg for PST biosynthesis. At the same time, α-ketoglutarate (generated by the TCA cycle) could also be catalyzed by alanine aminotransferase (ALT) and aspartate aminotransferase (AST) to produce Glu, in which genes encoding ALT (*alaat1*) and AST (*asp1*) both showed significant up-regulation. Glu could combine NH_3_ to produce Gln and further synthesize Arg in the presence of glutamine synthetase (GS), in which we found the up-regulation of the glutamine synthetase (GS)-encoding genes *glnA* and *glnA3*. The enhancement in Arg synthesis further supported the PST production increase in *A. tamarense* cells under NPs exposure (Figure 5 and Table S3).

Moreover, two genes encoding the heat shock proteins 70 (*hsp70*, *hsp70-5*) were up-regulated 2^2.75^- and 2^3.81^-folds, respectively. Heat shock proteins also act as antioxidants that could be stimulated in microalgal cells in response to high temperature, heavy metals and peroxide stress [30,31,32]. The proteasome pathway was significantly enriched in the KEGG analysis, and the related DEGs mainly included *psmd (2, 3, 12)*, *rpt4a* encoding regulatory elements, *psma (6, 7)* and *psmb (1, 3, 5, 6)* encoding core elements, which were all significantly up-regulated under the NPs exposure (Figure 5 and Table S3). The major function of proteasome is to degrade the misfolded or damaged proteins, thus maintaining the recycling of amino acids [33]. The biosynthesis of the antioxidant materials and PSTs required organic carbon and energy supply (mainly ATP); thus, we discussed the key important carbon metabolism processes in the next section.

### 2.7. Enhanced Carbon Metabolism Processes of A. tamarense under NPs Exposure

**Figure 6:**
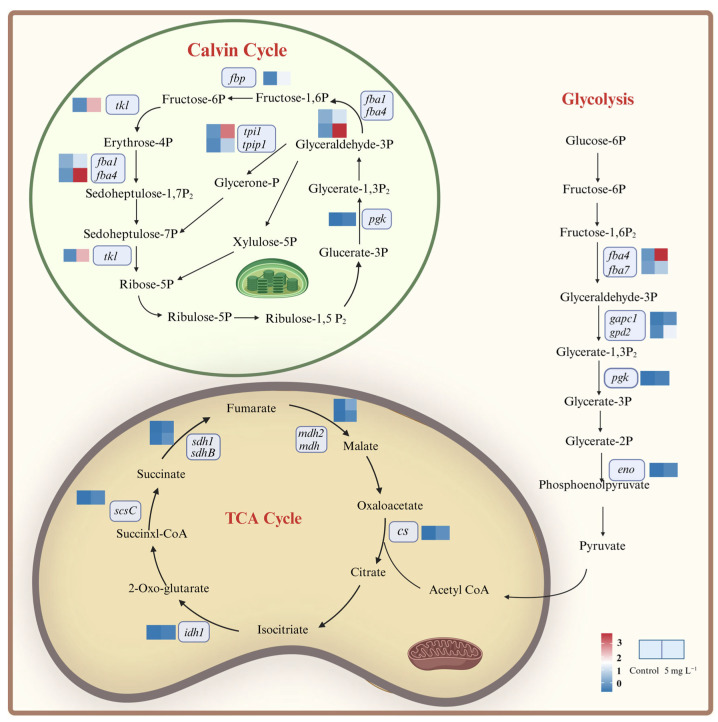
DEGs involved in the carbon metabolism processes in *A. tamarense* under PS NPs exposure.

Regarding the carbon metabolism processes, we mainly focused on the Calvin cycle, glycolysis and TCA cycle (Figure 6). Among them, the enhancement in the Calvin cycle could increase the carbon fixation ability of *A. tamarense* [34]. The gene (*pgk*) encoding phosphoglycerate kinase (PGK) up-regulated 2^3.09^-fold, which is responsible for the CO_2_ reduction reaction phase (Figure 6 and Table S3). Genes involved in the RuBP regeneration phase, such as *fba1* and *fba4* encoding aldolase (FBA), *fbp* encoding fructose-1,6-bisphosphatase (FBP), *tkl* encoding transketolase (TKL), *tpiA* and *tpip1* encoding propylphosphate isomerase (TPI) were significantly up-regulated, with the most significant up-regulation in *tkl* (up-regulated 2^3.15^-fold; Figure 6 and Table S3). The down-regulation of photosynthetic genes could lead to the insufficient supply of NADPH and ATP (Figure 4), and the up-regulation of the genes in the Calvin cycle could increase the conversion efficiency from light energy to carbohydrates, which might be a compensatory mechanism of *A. tamarense* cells under NPs exposure [35].

Under NPs exposure, most genes related to the glycolytic pathway were significantly up-regulated, including *fba4* and *fba7* encoding fructose 1,6-bisphosphate aldolase (FBA), *pgk* encoding phosphoglycerate kinase (PGK), *gapc1* and *gpd2* encoding glyceraldehyde-3-phosphate dehydrogenase (GAPD) and *eno* encoding enolase (Figure 6 and Table S3). The up-regulation of those genes could accelerate ATP and pyruvate production, and pyruvate could further form acetyl-CoA participating in various bioprocesses such as the TCA cycle and PST biosynthesis [36].

The TCA cycle is the final metabolic pathway for sugars, fats and proteins [37]. Citrate synthase (CS) and isocitrate dehydrogenase (IDH) are the most important rate-limiting enzymes in the TCA cycle, whose encoding genes *cs* and *idh1* were up-regulated by 2^4.66^- and 2^2.17^-folds in response to NPs exposure, respectively (Figure 6 and Table S3). Meanwhile, genes encoding succinyl CoA synthase (LSC; *scsC*), succinate dehydrogenase (SDH; *sdh1, sdhB*) and malate dehydrogenase (MDH; *mdh2, mdh*) were significantly up-regulated, which could lead to the acceleration of the TCA cycle (Figure 6 and Table S3). Each TCA cycle could produce large amounts of energy (GTP) as well as reducing power (NADH and FADH2), which could generate ATP through the oxidative phosphorylation process in the mitochondria. Starting from 1 molecule of glucose decomposed by glycolysis into 2 molecules of pyruvate in the TCA cycle, a total of 25 ATP molecules could be produced in the whole cycle [38]. Therefore, the TCA cycle is the major pathway for energy acquisition in microalgal cells. Many previous studies have indicated the enhancement in the TCA cycle in microalgal cells under environmental stress, such as high CO_2_, organic pollutants exposure, heavy metals, etc. [39,40,41]. For the MNPs studies, Li et al. (2023) [42], Sheng et al. (2023) [43] and Zhu et al. (2024) [44] also found the enhancement in the TCA cycle in *T. pseudonana*, *Heterosigma akashiwo* and *Chlorella pyrenoidosa*. Taken together, we know that an enhanced Calvin cycle could compensate the inhibition of the light reaction, and the enhanced glycolytic pathway together with the TCA cycle could provide more ATP to assist *A. tamarense* cells in resisting the NPs stress. Overall, some stress responses such as the down-regulation of genes encoding PS I and PS II showed the toxicity of NPs on *A. tamarense*, while the up-regulation of genes encoding antioxidant components and carbon metabolisms indicated the positive responses of *A. tamarense* under NPs exposure.

**Figure 7:**
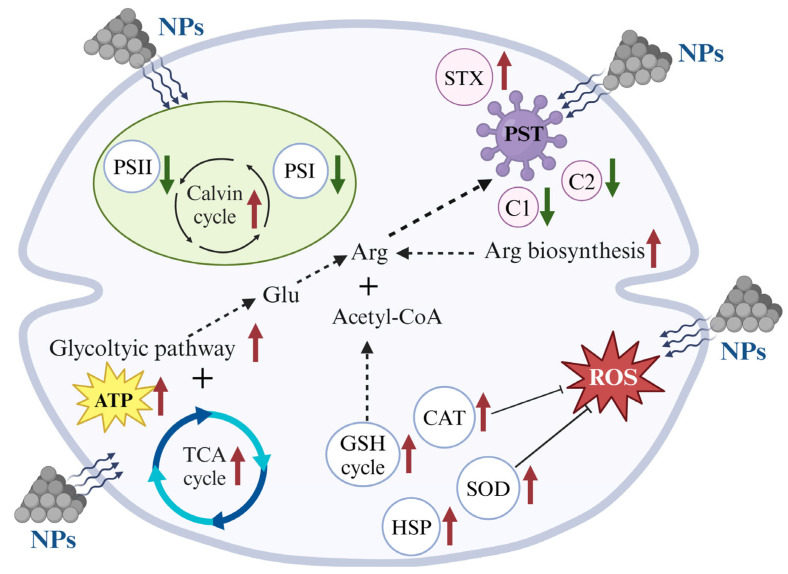
The possible response mechanisms of *A. tamarense* under NPs exposure.

Based on the physiological–biochemical and transcriptomic analysis, the responding mechanisms of *A. tamarense* triggered by NPs exposure could be summarized. Firstly, the NPs inhibited the photosynthetic efficiencies and caused cellular oxidative stress. Due to the small particle size, the photosynthetic inhibition was more likely due to light shading caused by NPs particles adsorbing on the microalgal cell surface and aggregating with microalgal cells [14,45]. Secondly, NPs exposure stimulated the production increase in PSTs, as well as increased STX proportions but decreased C2 proportions. One previous study applied the environmental-related PS MPs concentration (1 μm, 5 μg L^−1^) on *A. tamarense*, and their results showed 5 μg L^−1^ PS MPs also aroused the elevated cellular ROS and PST production, consistent with the results under the lab experimental MPs concentration (5 mg L^−1^) but much less significant [19]. In order to dig out more information on the genes controlling the physiological–biochemical performance, the lab toxic concentration of NPs (5 mg L^−1^) was applied in our study, which could provide a reference for the NPs affecting *A. tamarense* in natural marine environment. Lastly, the molecular mechanisms of NPs that induced a PST production increase could be concluded from the transcriptomic information as follows: an enhanced GSH cycle could provide the raw materials (Glu) of PSTs, together with the up-regulation of acetyl-CoA (another key raw material of PST biosynthesis) biosynthesis genes and enhanced TCA cycle (providing energy) (Figure 7).

## 3. Materials and Methods

### 3.1. The Cultivation of Experimental Microalgae

In this study, the *A. tamarense* strain (CCMA 118, marine algae of Xiamen University, Xiamen, China) was cultured by autoclaved natural seawater enriched with L1 medium. *A. tamarense* was cultured under laboratory-controlled conditions, which were 20 ± 1 °C, 100 μmol photons m^−2^ s^−1^ light intensity under 12 h:12 h light:dark cycles. The initial density of 10^3^ cells mL^−1^ was used in the following experiments. The microalgae culture was maintained at the exponential phase before the experiment.

### 3.2. NPs Exposure Experiments

The 100 nm polystyrene (PS) NPs for the experiments were purchased from Daekj Scientific Co., Ltd. (Tianjin, China), which were spherical solid microspheres and the average sizes of them were 0.1 ± 0.003 μm. The NPs particles were first dispersed in 10 mL deionized water (2.5%, *w*/*v*) as a working solution. To clarify the toxic mechanisms of 100 nm PS NPs on *A. tamarense* cells, 5 mg L^−1^ PS NPs concentration was chosen as the toxic concentration based on the results of previous studies [46], which was diluted from the working solution. The experimental culture volume was 1.5 L, one blank control without NPs was set up and each treatment had three replicates.

The experiment lasted 144 h, during which the microalgal cell densities, the PSII photosynthetic efficiencies, cellular pigment contents, cellular reactive oxygen species (ROS) levels, key components in the antioxidant system, including the activities of superoxide dismutase (SOD), catalase (CAT), glutathione reductase (GR), glutathione peroxidase (GPx) and the contents of malondialdehyde (MDA), glutathione (GSH) and oxidized glutathione (GSSG), as well as the PST contents and compositions, were measured at 48, 96 and 144 h. The samples for the transcriptional analysis were taken at 48 h.

### 3.3. The Measurements of Physiological and Biochemical Parameters

Microalgal cell density was counted by the hemocytometer using an optical microscope (40×; Olympus, Tokyo, Japan), and the relative growth rate (μ, day^−1^) was calculated by Equation (1):

μ = [ln (N_t_) − ln (N_0_)]/t (1)

where N_t_ is the cell density at t h and N_0_ is the cell density at 0 h.

Two PSII photosynthetic parameters were measured by a pulse amplitude-modulated (Water-PAM fluorometer, Walz, Effeltrich, Germany), including the maximum quantum yields of PSII (F_v_/F_m_) and maximum electron transportation rates (rETR_max_), which were generated from the Induction Curve (IC) and the Rapid Light Curve (RLC) [47]. An amount of 10 mL microalgal culture was dark-adapted for 20 min before measurement. The cellular chl *a* and total carotenoid contents were measured with a spectrophotometer (UV-8000, Metash, Shanghai, China) according to Parsons et al. (1984) [48].

Cellular ROS level was measured according to Zhao et al. (2020) [49], which was based on the reaction between 2′7′-Dichlorofluorescein diacetate (DCFH-DA) and ROS. The 50 mL microalgal cultures were centrifuged (10 min at 1000× *g*) and re-suspended in 1.5 mL sterilized seawater to obtain concentrated microalgal cells. An amount of 10 μM DCFH-DA was added to the concentrated culture and incubated in the dark at 20 °C for 60 min. After that, the mixture was centrifuged (1000× *g* for 10 min) and washed three times with PBS buffering solution (pH = 7.5), followed by resuspension in 0.5 mL of PBS buffering solution. The fluorescence intensity was quantified by a multi-mode microplate reader (2300, PerkinElmer Inc., Waltham, MA, USA) and the ROS levels were shown as DCF fluorescence per 10^5^ *A. tamarense* cells.

Two hundred and fifty milliliter microalgal cultures were centrifuged for 15 min (3000× *g*, 4 °C) and re-suspended in one mL PBS buffering solutions. The mixture was disrupted by a JY92-II ultrasonic cell pulverizer (NingBo Scientz Biotechnology Co., Ltd., Ningbo, China) and centrifuged (3000× *g*, 15 min, 4 °C), then the supernatant was used to measure the activities of SOD, CAT, GR, GPx and the contents of MDA, GSH and GSSG (detailed information was shown in Text S1). The enzymatic activities and substance concentrations were normalized by the total soluble protein concentrations.

### 3.4. Measurements of Cellular PST Contents and Compositions

The 100 milliliter microalgal cultures were filtered on GF/F filters (25 mm, Whatman, Germany) and preserved at −20 °C. Microalgal cells on the filters were eluted by acetic acid (5 mL, 0.1 mol L^−1^) twice, then were centrifuged for 5 min at 8000× *g* to extract cellular PSTs. The supernatants were transferred into 10 mL sterile centrifuge tubes and analyzed by high-performance liquid chromatography (HPLC) [50]. The HPLC settings and measuring progresses are shown in the Supplementary Materials (Text S2).

### 3.5. Transcriptomic Analysis

At 48 h, 1 L microalgal cultures from each treatment were filtered on 1.2 μm cellulose acetate membranes filters (Shanghai Xinya, Shanghai, China) and stored at −80 °C for the transcriptional analysis. The transcriptional analysis processes and data processing are shown in Text S3.

### 3.6. Statistical Analysis

All figures were conducted by Origin 2023b (OriginLab, Northampton, MA, USA), and the data were presented as means ± standard deviations (S.D.). The statistical differences between the control and the NPs group were evaluated by one-way ANOVA tests followed by Student’s *t*-tests using SPSS 25.0 (SPSS Inc., Chicago, IL, USA). *p* < 0.05 was considered as statistically significant.

## 4. Conclusions

This study comprehensively elucidated the responding mechanisms of 100 nm PS NPs on *A. tamarense* cells based on physiological–biochemical performance and transcriptional analysis. Inhibition of population growth and photosynthetic efficiencies of *A. tamarense* were found under NPs exposure, corresponding to the increase in the cellular total carotenoids and PSTs, as well as the activation of the antioxidant system. The gene expression changes support the physiological–biochemical results but provide more comprehensive gene information, which could be concluded as follows: (i) down-regulation of genes encoding light reaction centers (PSI and PSII) could be compensated by the up-regulation of genes encoding the Calvin cycle, ensuring the organic carbon production; (ii) the activated antioxidant components on the transcriptional level including SOD, CAT, GSH cycle and HSP; (iii) enhanced glycolytic pathway together with the TCA cycle could provide more ATP for *A. tamarense* cells resisting the NPs stress; (iiii) more Glu and acetyl-CoA produced by the enhanced GSH cycle and glycolytic pathway, respectively, as well as the up-regulation of Glu and Arg biosynthesis genes supported the increase in PST contents under NPs exposure.

## Figures and Tables

**Table 1:** The relative growth rate, maximum quantum yields of PSII (Fv/Fm), maximum electron transportation rates (rETR_max_), cellular chlorophyll (chl *a*) and the total carotenoid contents of *A. tamarense* in the control and under NPs exposure. Data are shown as mean ± S.D. * indicates a statistical significance compared to the controls at *p* < 0.05 level, ** indicates *p* < 0.01 level.

	Treatments	48 h	96 h	144 h
Relative growth rate (d^−1^)	Control	0.46 ± 0.02	0.64 ± 0.10	0.18 ± 0.01
	5 mg L^−1^	0.33 ± 0.05 **	0.72 ± 0.06	0.15 ± 0.04
F_v_/F_m_	Control	0.54 ± 0.08	0.53 ± 0.05	0.51 ± 0.02
	5 mg L^−1^	0.52 ± 0.01	0.52 ± 0.03	0.51 ± 0.05
rETR_max_ (μmol electrons m^−2^s^−1^)	Control	64.21 ± 1.21	58.40 ± 1.56	55.20 ± 4.01
	5 mg L^−1^	60.10 ± 1.90 *	56.10 ± 1.28	51.32 ± 2.10
Chl *a* (pg cell^−1^)	Control	1.91 ± 0.11	2.00 ± 0.04	2.52 ± 0.03
	5 mg L^−1^	2.00 ± 0.06	2.04 ± 0.02	2.60 ± 0.15
Total carotenoids (pg cell^−1^)	Control	1.43 ± 0.02	1.50 ± 0.06	1.52 ± 0.07
	5 mg L^−1^	1.50 ± 0.05	1.82 ± 0.09 **	1.94 ± 0.06 **

## Data Availability

Data is contained within the article and Supplementary Materials.

## Supplementary Materials

The following supporting information can be downloaded at: https://www.mdpi.com/article/10.3390/ijms25137297/s1.
