# Supplementary material for: The Effecting Mechanisms of 100 nm Sized Polystyrene Nanoplastics on the Typical Coastal Alexandrium tamarense
Source: Int J Mol Sci. 2024 Jul 2;25(13):7297. doi: 10.3390/ijms25137297 (PMC11242399; doi:10.3390/ijms25137297)
Supplement: Supplementary file 1 [file ijms-25-07297-s001.zip › ijms-3040688-supplementary.pdf]

Supplemental Material

The effecting mechanisms of 100 nm sized polystyrene nanoplastics on the typical coastal *Alexandrium tamarense*

Luying Li [1,2#], Qian Liu[3,4#], Bo Li[1]*, Yan Zhao[2]

[1]Marine Science and Technology College, Zhejiang Ocean University, Zhoushan, 316022 China

[2] College of Marine Life Sciences, Department of Marine Ecology, Ocean University of China, Qingdao, China 266003

[3] Marine Science Research Institute of Shandong Province, Qingdao, China, 266104

[4] Qingdao Key Laboratory of Coastal Ecological Restoration and Security, Qingdao, China， 266104

Supporting Information I: Materials and Methods

Text S1. Determination of antioxidant enzyme activities and antioxidant substances contents.

Text S2. HPLC systems and PSTs standards.

Text S3. Transcriptomic analysis.

Supporting Information II: Tables

Table S1. Statistical results of sequencing of *A. tamarense* in control group and 5 mg L^{-1} group at 48 h.

Table S2. Overview of Trinity assembly results *A. tamarense* in control group and 5 mg L^{-1} group at 48 h.

Table S3. The expression level and information of genes in *A. tamarense* under 5 mg L^{-1} group at 48 h.

Text S1. Determination of antioxidant enzyme activities and antioxidant substances contents

The determination of Superoxide dismutase (SOD), Catalase (CAT), Glutathione (GSH) and glutathione reductase (GR), glutathione peroxidase (GPx) were carried out using assay kits purchased from Nanjing Jiancheng Company (Nanjing Jiancheng Biology Research Institute, Nanjing, China), and the contents of MDA, GSH, and GSSG were also measured by their corresponding assay kits (Beyotime Institute of Biotechnology, Shanghai, China). The intracellular total soluble proteins were measured by a Pierce BCA Protein Assay Kit (Thermo Scientific, USA).

Text S2. HPLC systems and PSTs standards

The HPLC system is composed of a Waters e2695 separation module, a post-column reaction module, and a 2475 fluorescence detector (Waters Corporation, Milford, MA, USA). The separation module includes a quaternary pump, an autosampler and a column heater. The post-column reaction module includes a double-head pump to deliver oxidant and acid solutions, and a reaction unit for temperature control. The system was operated with the software Empower 2. PSTs standards (C1/2, GTX1/4, GTX 2/3, GTX5, GTX6, NEO, STX, and dcSTX) were obtained from the Certified Reference Materials Program of the National Research Council (Halifax, Nova Scotia, Canada, https://nrc.canada.ca/en/research-development/products-services/shop-nrc), and the limits of detection were 1 ng mL^{-1} of all PSTs standards.

HPLC method A: Separation of carbamate and decarbamoyl toxins (GTX1/4, GTX2/3, NEO, STX, dcSTX) was performed on a reverse-phased Zorbax Bonus-RP column (3.5 μm, 150 × 4.6 mm i.d., Agilent Technologies, USA) equipped with a guard column. Toxins were eluted with a phosphoric acid buffer system containing sodium heptanesulfonate using a gradient method. Two mobile phases were used: mobile phase A was an ammonium phosphate buffer (5.5 mmol L^{-1}, pH 7.1) with 11 mmol L^{-1} sodium heptanesulfonate as an ion-pair reagent, and mobile phase B was an ammonium phosphate buffer (16.5 mmol L^{-1}, pH 7.1) with 11 mmol L^{-1} sodium heptanesulfonate and 11.5% acetonitrile. Toxins were eluted with mobile phase A from 0 to 11.4 min, mobile phase B from 11.5 to 22 min, and mobile phase A again from 22.1 to 29 min. The flow rate of both mobile phases was 0.8 mL min^{-1}.

HPLC method B: A Synergi Hydro-RP column (4 μm, 150 × 4.6 mm i.d., Phenomenex, USA) equipped with a guard column was used for the analysis of N-sulfocarbamoyl toxins (C1/2). Two mobile phases were adopted. Mobile phase A was

an ammonium solution (1%, pH 5.8), with 3 mmol L^{-1} tetrabutyl ammonium phosphate as the ion-pair reagent. Mobile phase B was an ammonium solution (1%, pH 5.8) with 3 mmol L^{-1} tetrabutyl ammonium phosphate and 4% acetonitrile. N-sulfocarbamoyl toxins were eluted with mobile phase A from 0 to 8.0 min, mobile phase B from 8.1 to 9.1 min, and mobile phase A again from 9.1 to 16.0 min. The flow rate of the two mobile phases was 0.8 mL min^{-1}.

The oxidant solution was 5 mmol L^{-1} H_5IO_6 aqueous solution (with pH adjusted to 7.8 with 5 mmol L^{-1} NaOH), and the acid solution was 0.75 mol L^{-1} HNO_3. The temperature for the post-column reaction was controlled at 85°C. The excitation and the emission wavelength for PST detection were set to 330 nm and 390 nm, respectively. During the analysis process, we re-injected the mix standards every 8 - 10 samples, and quality control was carried out by determining the changes of the mix standards throughout the analysis process, and the content changes did not exceed ± 20%.

Text S3. Transcriptomic analysis

After 48 h exposure, the algal samples selected for RNA sequencing including: Control group and 5 mg L^{-1} group. Total RNA of each sample was extracted using TRIzol (Invitrogen Life Technologies, Carlsbad, USA) according to the manufacturer's instructions. The quality and integrality of the total RNA were assessed using the ratio of A260/280 and electrophoresed agarose gel. The mRNA samples were prepared for sequencing using Illumina HiSeqTM 2500 by Lianchuan Biotechnology Co. (Hangzhou, China). After removal of the reads that contained adapters, poly-N strands and low-quality reads, the processed clean data were upload to the NCBI (https://www.ncbi.nlm.nih.gov/). The expression level for each gene was calculated by Fragments Per Kilobase of transcript per Million mapped reads (FPKM). Differentially expressed genes (DEGs) between two different groups were calculated using DESeq (v1.16), while genes with p-values less than 0.05 and $\log_2$(fold change) values ($\log_2$FC values) larger than 1 were considered as differentially expressed genes (DEGs). All DEGs were functionally annotated by the non-redundant protein (NR) database. Genes were also carried to classification and enrichment analysis of the Gene ontology (GO) and Kyoto Encyclopedia of Genes and Genomes (KEGG), respectively. Principal component analysis (PCA) was performed with R package gmodels (http://www.r-project.org/) for the RNA sequencing data set.

Table S1. Statistical results of sequencing of *A. tamarense* in control group and 5 mg L^{-1} group at 48 h.

Sample	Raw Reads	Valid Reads	Valid%	Q20%	Q30%	GC%
Control-1	58280132	57290968	98.30	98.75	95.90	59.44
Control-2	56575846	55628108	98.32	98.74	95.87	59.44
Control-3	57073370	56219574	98.50	98.73	95.83	59.33
5 mg L^{-1}-1	57123032	55495696	97.15	98.72	95.90	59.97
5 mg L^{-1}-2	56776308	54988092	96.85	98.73	95.89	60.12
5 mg L^{-1}-3	51156000	49418798	96.60	98.67	95.73	59.95

Table S2. Overview of Trinity assembly results *A. tamarense* in control group and 5 mg L^{-1} group at 48 h.

Index	Transcript	Gene
All	134969	90382
GC%	62.00	62.26
Min Length	201	201
Median Length	726	764
Max Length	17791	17791
Total Assembled Base	130946496	90006258
N50	1472	1540

Table S3. The expression level and information of genes in *A. tamarense* under 5 mg L-1 group at 48 h.

Gene ID	Gene name	Annotation	Log_2 (Fold Change)
**Photosynthesis**			
TRINITY_DN7387_c0_g1	*psbE*	Cytochrome b559 alpha subunit of photosystem II	-5.76
TRINITY_DN41744_c2_g3	*psbB*	Photosystem II CP47 chlorophyll apoprotein	-5.81
TRINITY_DN42453_c1_g1	*psbA*	D1 reaction center protein of photosystem II	-5.16
TRINITY_DN41169_c0_g1	*psbD*	D2 reaction center protein of photosystem II	-4.74
TRINITY_DN39023_c0_g2	*psbC*	Photosystem II 44 kDa protein (chloroplast)	-5.00
TRINITY_DN43742_c0_g2	*psaB*	Photosystem I P700 chlorophyll a apoprotein A2	-5.71
TRINITY_DN43742_c0_g3	*psaA*	P700 apoprotein A1 of photosystem I	-4.61
TRINITY_DN41169_c1_g16	*petD*	Subunit IV of cytochrome b6/f complex	-4.84
TRINITY_DN41169_c1_g5	*petB*	Cytochrome b6 (plastid)	-4.66
TRINITY_DN43286_c1_g1	*atpA*	CF1 alpha subunit of ATP synthase	-5.69
**Carbon fixation in photosynthetic organisms**			
TRINITY_DN43111_c1_g15	*pgk*	Phosphoglycerate kinase	3.09
TRINITY_DN42228_c0_g11	*fba1*	Fructose-bisphosphate aldolase	1.04
TRINITY_DN43354_c0_g12	*fba4*	Fructose-bisphosphate aldolase	3.1
TRINITY_DN43631_c0_g3	*fbp*	Fructose-1,6-bisphosphatase	3.08
TRINITY_DN24323_c0_g1	*tkl*	Transketolase	3.15
TRINITY_DN2424_c0_g1	*tpiA*	Triosephosphate isomerase	3.01
TRINITY_DN40197_c0_g14	*tpip1*	Triosephosphate isomerase	1.88
**Porphyrin and chlorophyll metabolism**			
TRINITY_DN37973_c0_g1	*chlH*	Magnesium chelatase subunit	2.41

TRINITY_DN44335_c0_g8	*pora*	Protochlorophyllide reductase	1.03
TRINITY_DN38982_c0_g22	*hemE*	Uroporphyrinogen decarboxylase	1.02
TRINITY_DN40219_c1_g13	*eprs*	Glutamyl-tRNA synthetase	2.36
**Glutathione metabolism**			
TRINITY_DN26235_c0_g1	*gpx6*	Glutathione peroxidase	2.11
TRINITY_DN39480_c2_g1	*gpxmc1*	Glutathione peroxidase	3.35
TRINITY_DN40251_c0_g1	*gor*	Glutathione reductase	2.45
TRINITY_DN42559_c1_g8	*dhar1*	Dehydroascorbate reductase	1.07
TRINITY_DN21271_c1_g1	*dhar2*	Dehydroascorbate reductase	1.08
TRINITY_DN40909_c0_g2	*gst-7*	Glutathione S-transferase	1.07
TRINITY_DN42785_c0_g4	*gstp1*	Glutathione S-transferase	4.84
**Glycolysis / Gluconeogenesis**			
TRINITY_DN43354_c0_g12	*fba4*	Fructose-bisphosphate aldolase	3.1
TRINITY_DN38771_c1_g26	*fba7*	Fructose-bisphosphate aldolase	1.02
TRINITY_DN2424_c0_g1	*tpiA*	Triosephosphate isomerase	3.01
TRINITY_DN40197_c0_g14	*tpip1*	Triosephosphate isomerase	1.88
TRINITY_DN40310_c0_g5	*pgk*	Phosphoglycerate kinase	2.39
TRINITY_DN32450_c0_g1	*gapc1*	Glyceraldehyde-3-phosphate dehydrogenase	1.27
TRINITY_DN45279_c0_g15	*gpd2*	Flyceraldehyde-3-phosphate dehydrogenase	2.30
TRINITY_DN45000_c1_g7	*eno*	Enolase	2.53
**Citrate cycle (TCA cycle)**			
TRINITY_DN25082_c0_g1	*cs*	Citrate synthase	4.66
TRINITY_DN37844_c0_g3	*idh1*	Isocitrate dehydrogenase	2.17
TRINITY_DN10451_c0_g1	*scsC*	Succinate-CoA ligase (LSC)	2.97
TRINITY_DN43328_c0_g14	*sdh1*	Succinate dehydrogenase (SDH)	3.63

TRINITY_DN45147_c1_g7	*sdhB*	Succinate dehydrogenase	2.77
TRINITY_DN38955_c0_g1	*mdh2*	Malate dehydrogenase	4.38
TRINITY_DN43276_c1_g19	*mdh*	Malate dehydrogenase	3.52
**Superoxide dismutase**			
TRINITY_DN7230_c0_g1	*sod1*	Superoxide dismutase	3.21
TRINITY_DN37239_c0_g2	*sodb*	Superoxide dismutase	2.75
TRINITY_DN37992_c0_g2	*sod2*	Superoxide dismutase	2.91
**Catalase**			
TRINITY_DN16364_c0_g1	*cat*	Catalase	3.75
**Heat shock reaction**			
TRINITY_DN45028_c2_g19	*hsp70-5*	Heat shock 70 kDa protein	3.81
TRINITY_DN43336_c2_g1	*hsp70*	Heat shock 70 kDa protein	2.75
**Protesome**			
TRINITY_DN38711_c0_g1	*psmd2*	Regulatory proteasome non-atpase subunit	2.89
TRINITY_DN39016_c1_g1	*psmd12*	Regulatory proteasome non-atpase subunit	2.79
TRINITY_DN38598_c1_g1	*psmd3*	Regulatory proteasome non-atpase subunit	2.09
TRINITY_DN36657_c0_g2	*rpt4a*	Regulatory proteasome non-atpase subunit	2.27
TRINITY_DN48524_c0_g1	*psma7*	Proteasome subunit alpha	2.24
TRINITY_DN42330_c1_g21	*psma6*	Proteasome subunit alpha	2.48
TRINITY_DN39650_c1_g9	*psmb1*	Proteasome subunit beta	1.97
TRINITY_DN40437_c0_g6	*psmb6*	Proteasome subunit beta	2.20
TRINITY_DN33440_c0_g1	*psmb5*	Proteasome subunit beta	2.23
TRINITY_DN38601_c0_g13	*psmb3*	Proteasome subunit beta	2.41
**PSTs production**			
TRINITY_DN37681_c0_g3	*sahh*	S-adenosylhomocysteine hydrolase	2.62

TRINITY_DN42617_c0_g7	*map2b*	Methionine aminopeptidase	2.15
TRINITY_DN32694_c0_g1	*metK*	S-adenosylmethionine synthetase	2.12
**Arginine biosynthesis**			
TRINITY_DN44903_c0_g4	*glnA*	Glutamine synthetase	1.02
TRINITY_DN37974_c0_g1	*glnA3*	Glutamine synthetase	2.04
TRINITY_DN39023_c0_g6	*alaat1*	Glanine transaminase	3.84
TRINITY_DN39837_c1_g7	*asp1*	Aspartate aminotransferase	2.78
